# Genetic Predispositions Between COVID-19 and Three Cardio-Cerebrovascular Diseases

**DOI:** 10.3389/fgene.2022.743905

**Published:** 2022-03-16

**Authors:** Jiang-Shan Tan, Ningning Liu, Ting-Ting Guo, Song Hu, Lu Hua, Qiujin Qian

**Affiliations:** ^1^ State Key Laboratory of Cardiovascular Disease, Thrombosis Center, National Clinical Research Center of Cardiovascular Diseases, Fuwai Hospital, National Center for Cardiovascular Diseases, Chinese Academy of Medical Sciences and Peking Union Medical College, Beijing, China; ^2^ Peking University Sixth Hospital/Institute of Mental Health, Beijing, China; ^3^ NHC Key Laboratory of Mental Health (Peking University), National Clinical Research Center for Mental Disorders (Peking University Sixth Hospital), Beijing, China

**Keywords:** COVID-19, atrial fibrillation, ischemic stroke, coronary artery disease, Mendelian randomization

## Abstract

**Aims**: This study was aimed to apply a Mendelian randomization design to explore the causal association between coronavirus disease 2019 (COVID-19) and three cardio-cerebrovascular diseases, including atrial fibrillation, ischemic stroke, and coronary artery disease.

**Methods**: Two-sample Mendelian randomization was used to determine the following: 1) the causal effect of COVID-19 on atrial fibrillation (55,114 case participants vs 482,295 control participants), coronary artery disease (34,541 case participants vs 261,984 control participants), and ischemic stroke (34,217 case participants vs 40,611 control participants), which were obtained from the European Bioinformatics Institute, and 2) the causal effect of three cardio-cerebrovascular diseases on COVID-19. The single-nucleotide polymorphisms (SNPs) of COVID-19 were selected from the summary-level genome-wide association study data of COVID-19-hg genome-wide association study (GWAS) meta-analyses (round 5) based on the COVID-19 Host Genetics Initiative for participants with European ancestry. The random-effects inverse-variance weighted method was conducted for the main analyses, with a complementary analysis of the weighted median and Mendelian randomization (MR)-Egger approaches.

**Results**: Genetically predicted hospitalized COVID-19 was suggestively associated with ischemic stroke, with an odds ratio (OR) of 1.049 [95% confidence interval (CI) 1.003–1.098; *p* = 0.037] in the COVID-19 Host Genetics Initiative GWAS. When excluding the UK Biobank (UKBB) data, our analysis revealed a similar odds ratio of 1.041 (95% CI 1.001–1.082; *p* = 0.044). Genetically predicted coronary artery disease was associated with critical COVID-19, with an OR of 0.860 (95% CI 0.760–0.973; *p* = 0.017) in the GWAS meta-analysis and an OR of 0.820 (95% CI 0.722–0.931; *p* = 0.002) when excluding the UKBB data, separately. Limited evidence of causal associations was observed between critical or hospitalized COVID-19 and other cardio-cerebrovascular diseases included in our study.

**Conclusion**: Our findings provide suggestive evidence about the causal association between hospitalized COVID-19 and an increased risk of ischemic stroke. Besides, other factors potentially contribute to the risk of coronary artery disease in patients with COVID-19, but not genetics.

## Introduction

The coronavirus disease 2019 (COVID-19) pandemic is caused by severe acute respiratory syndrome coronavirus 2 (SARS-CoV-2) and is rapidly evolving as a major threat to global health. As of December 11, 2021, the COVID-19 pandemic has led to more than 269 million confirmed cases with more than 5.3 million deaths. The pathophysiology of SARS-CoV-2 is characterized by the overproduction of inflammatory cytokines (such as IL-6 and TNF-α) (Ye et al., 2020), which might result in systemic inflammation, acutely affecting the cardiovascular system (Azevedo et al., 2021). The observational association between cardio-cerebrovascular diseases (including atrial fibrillation, ischemic stroke, and coronary artery disease) and COVID-19 has been described in many previous studies (Zuin et al., 2020; Azevedo et al., 2021). However, these findings may have been confounded by some unmeasured risk factors, and uncertainties remain about the causal association between COVID-19 and these cardio-cerebrovascular conditions.

Mendelian randomization (MR) is a technique that has recently emerged and utilizes genetic variants of risk factors as instruments to assess the causality between the risk factor and a particular disease (Smith and Ebrahim, 2003; Davey Smith and Hemani, 2014; Davies et al., 2018). It is conceptually similar to prospective randomized controlled trials (RCTs), although MR may be conducted retrospectively. Since all inherited genetic variants are determined at conception, which occurs prior to disease onset, MR can avoid the potential bias of non-differential measurement error or confounding (Emdin et al., 2017; Tan et al., 2021a).

In the present study, we aimed to explain the observational association between COVID-19 and three cardio-cerebrovascular diseases by using bidirectional two-sample MR.

## Material and Methods

### Overall Study Design

We obtained summary data from previously published studies, which were approved by the institutional review committee in their respective studies. Therefore, no further sanction was required. We used bidirectional two-sample MR (Lawlor, 2016; Richmond et al., 2016) to assess the causal association between COVID-19 and cardiovascular conditions. First, we tested the effects of COVID-19 on three cardio-cerebrovascular diseases and then the causal effects of the three cardio-cerebrovascular diseases on COVID-19.

### Data Sources

#### Cardiovascular Disease

To identify relatively more independent genome-wide significant single-nucleotide polymorphisms (SNPs), the following criteria were used to filter our genetic instruments: 1) SNPs at a genome-wide significance threshold (*p* < 5.0 × 10^−7^) were included with clumping to ensure independence between SNPs (clumping *r* ^2^ cutoff = 0.001 and clumping window=10,000 kb) (Hemani et al., 2018; Tan et al., 2021b) were excluded; and 2) only SNPs that were available in both the exposure and outcome genome-wide association study (GWAS) datasets were selected in the present analysis. Other MR studies have used a similar MR method to relax the statistical threshold for genetic instruments once a few significant SNPs are available (Gage et al., 2017; Choi et al., 2019).

Corresponding data for cardiovascular diseases were obtained from the European Bioinformatics Institute and are available at https://www.ebi.ac.uk/gwas/downloads. To determine the bidirectional causal associations between COVID-19 and cardiovascular conditions, only cardiovascular disease patients whose SNP(s) were greater than or equal to 3 were included in this study. Thus, herein, we analyzed three cardio-cerebrovascular diseases, including atrial fibrillation (Roselli et al., 2018), coronary atrial diseases (van der Harst and Verweij, 2018), and ischemic stroke (Malik et al., 2018). Detailed information can be seen in Supplementary Table S1.

#### COVID-19

The SNPs were obtained from summary-level GWAS data of COVID-19-hg GWAS meta-analyses (release 5) based on the COVID-19 Host Genetics Initiative (2020) for participants with European ancestry (Supplementary Tables S2–S3), which was released on January 18, 2021 and was also made publicly available (COVID19-hg, 2021). All the GWAS data were the largest and most updated when we conducted this analysis.

Fourteen studies focused on the very severe respiratory confirmed COVID-19 cases, with a total of 1,388,342 participants (5,101 cases and 1,383,241 controls). When excluding the UK Biobank data, there were a total of 1,059,456 participants (4,792 cases and 1,054,664 controls). Very severe respiratory confirmed COVID-19 cases were defined as requiring hospitalization for laboratory-confirmed SARS-CoV-2 infection with death or respiratory support (COVID19-hg, 2021).

As a supplementary analysis, we also obtained SNPs from hospitalized COVID-19 patients and non-hospitalized COVID-19 patients including 12 studies, with a total of 16,645 participants (4,829 hospitalized cases and 11,816 controls). When excluding the UK Biobank data, a total of 10,363 participants (3,159 hospitalized cases and 7,204 controls) were selected in the present study. The meta-analysis of COVID-19 was performed with fixed-effects inverse-variance weighting. The results are available in genome builds 38. An allele frequency filter of 0.001 and an INFO filter of 0.6 were applied to each study before meta-analysis.

As described previously, we used independent clumped SNPs that met a threshold (*r* ^2^ < 0.001 and *p* < 5 × 10^−7^) as instrumental variables.

### Statistical Analysis

Because no individual-level GWAS data were available, we leveraged the recently developed method of two-sample MR analyses to assess the bidirectional causal association between COVID-19 and three cardio-cerebrovascular diseases, as described previously (Burgess et al., 2013).

Inverse-variance weighted (IVW) meta-analysis with a random-effects model was used in the principal analyses to combine the instrumental variable-ratio estimates across the associated SNPs (Larsson et al., 2020) and account for correlations between genetic variants. To avoid horizontal pleiotropy, we performed two sensitivity analyses. In the first sensitivity analysis, the weighted median approach was applied, in which valid estimates can be provided if there is more than 50% of the information coming from SNPs that are valid instrumental variables (Burgess et al., 2017). As a fundamental assumption in an MR analysis, we needed to ensure that the instrumental variables were associated with the outcome of our study only through the risk factor and not *via* any other causal pathway, which is so-called pleiotropy. Therefore, the MR-Egger method was used to estimate the directional pleiotropy (Bowden et al., 2015) in the second sensitivity analysis. The abovementioned analytical method is based on different models of horizontal pleiotropy. The value in us comparing the consistency through three different methods was to make our results more reliable (Burgess et al., 2015; Xu et al., 2017).

Finally, to assess the robustness of significant results, modified Cochran *Q* statistics were conducted to detect heterogeneous outcomes. Two-tailed *p* < .05 was used in all statistical tests. Bonferroni-corrected analysis was used with a threshold of *p* < .017 (*a* = 0.05/3 outcomes). Associations with *p* values between 0.017 and 0.05 were considered suggestive evidence of associations, requiring further confirmation.

The statistical coding and related data can be obtained from the corresponding author based on reasonable requests. All statistical analyses were performed using R version 4.0.3 (2020-10-10) (The R Foundation for Statistical Computing, Vienna, Austria) and the MR software packages (Verbanck et al., 2018; Broadbent et al., 2020).

## Results

### Genetic Instrumental Variables for COVID-19 and the Three Cardio-Cerebrovascular Diseases

As shown in Supplementary Tables S4–S10, we presented all genetic instruments associated with COVID-19 at a genome-wide significance level (*p* < 5 × 10^−7^) and all genetic instruments associated with the three cardio-cerebrovascular diseases at a genome-wide significance level (*p* < 5 × 10^−8^). Modified Cochran *Q* statistic revealed no notable heterogeneity across instrument SNP effects.

### The Effects of COVID-19 on the Three Cardio-Cerebrovascular Diseases

**FIGURE 1:**
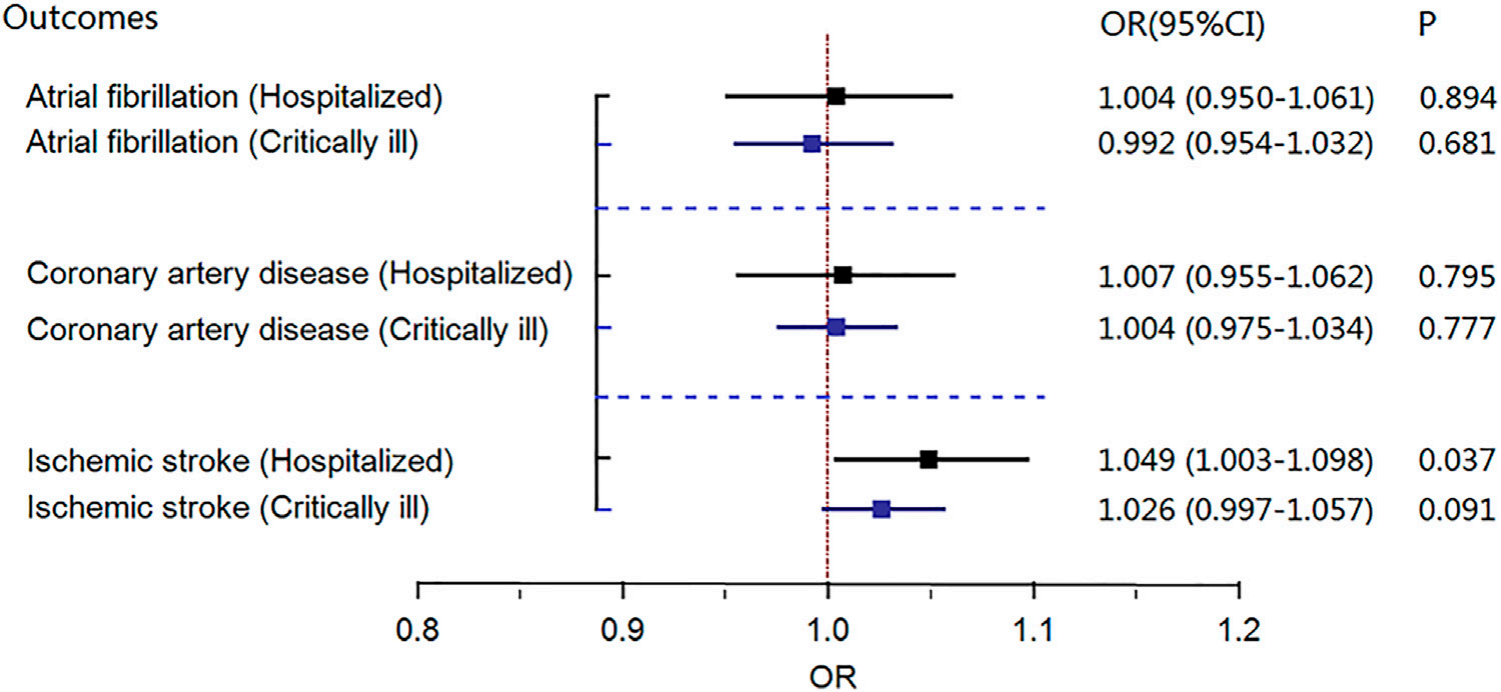
Results of the random effects Mendelian randomization inverse-variance weighted (MR IVW) model investigating the causal association between gene-predicted severe coronavirus disease 2019 (COVID-19) and risk of three cardiovascular diseases in COVID-19 genome-wide association study (GWAS) data. CI, confidence interval; OR, odds ratio; MR, Mendelian randomization; COVID-19, severe coronavirus disease 2019.

**FIGURE 2:**
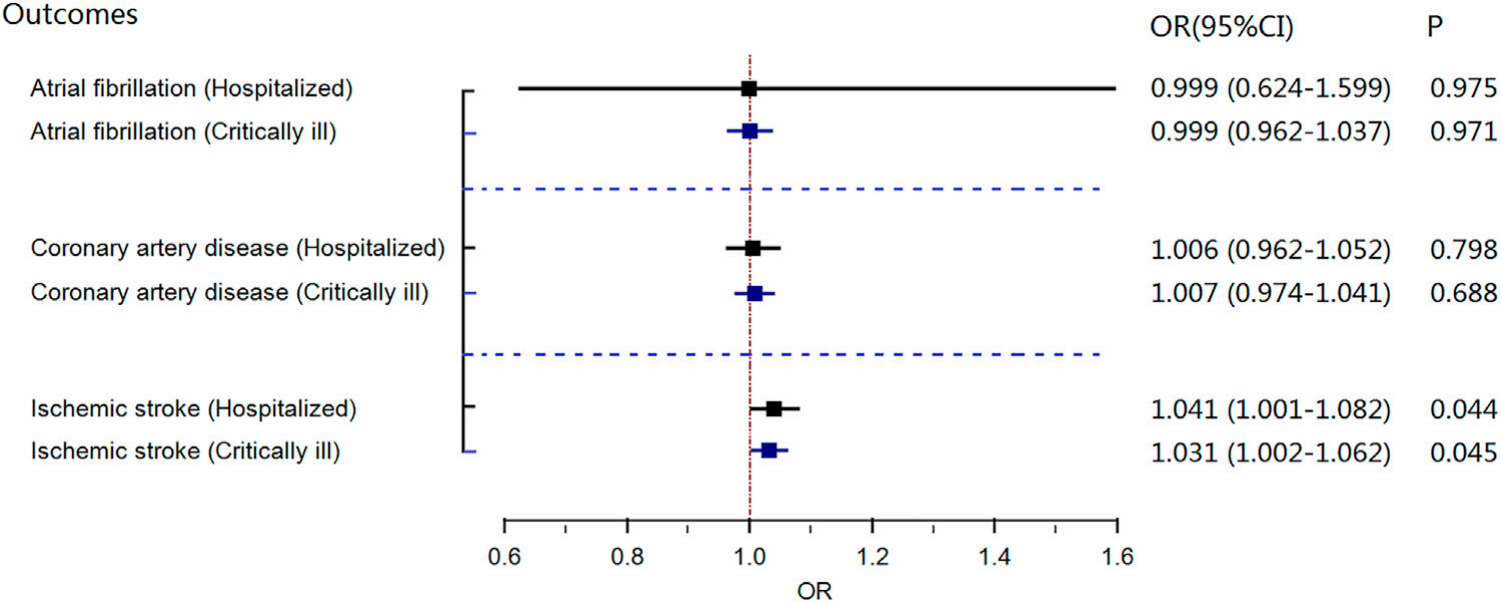
Results of the random-effects MR IVW model investigating the causal association of gene-predicted severe coronavirus disease 2019 (COVID-19) and risk of three cardiovascular diseases in COVID-19 GWAS data (without UKBB data). CI, confidence interval; OR, odds ratio; MR, Mendelian randomization; COVID-19, severe coronavirus disease 2019.

Genetically predicted hospitalized COVID-19 was suggestively associated with ischemic stroke in the COVID-19 Host Genetics Initiative genome-wide association study [random-effects MR IVW model, odds ratio (OR) = 1.049, 95% confidence interval (CI) = 1.003–1.098; *p* = .037, Figure 1], which was consistent with the results of GWAS meta-analysis (without the UKBB data), with an OR of 1.041 (random-effects MR IVW model, 95% CI 1.001–1.082; *p* = 0.044, Figure 2). There was no evidence supporting a causal association of COVID-19 with the risk of atrial fibrillation or coronary atrial diseases.

### The Effects of the Three Cardio-Cerebrovascular Diseases on COVID-19

**FIGURE 3:**
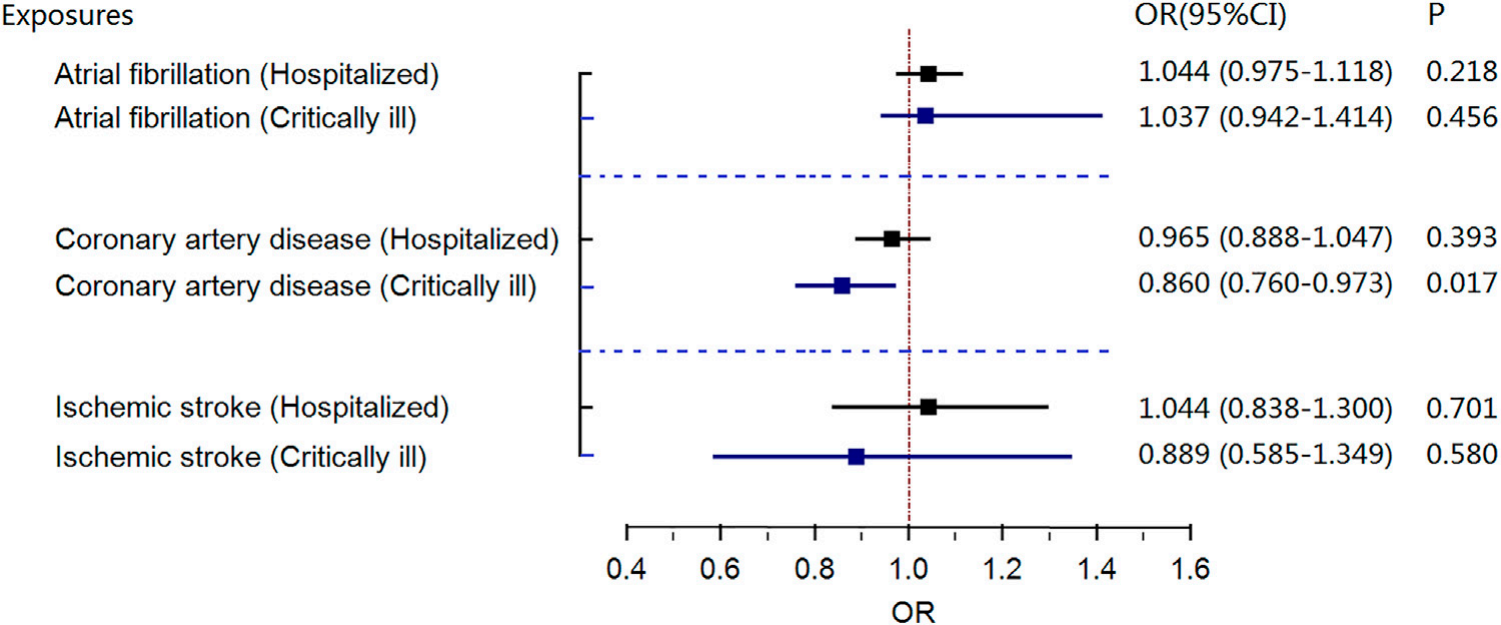
Results of the random-effects MR IVW model investigating the causal association between gene-predicted three cardiovascular diseases and risk of severe coronavirus disease 2019 (COVID-19) in COVID-19 GWAS data. CI, confidence interval; OR, odds ratio; MR, Mendelian randomization; COVID-19, severe coronavirus disease 2019.

**FIGURE 4:**
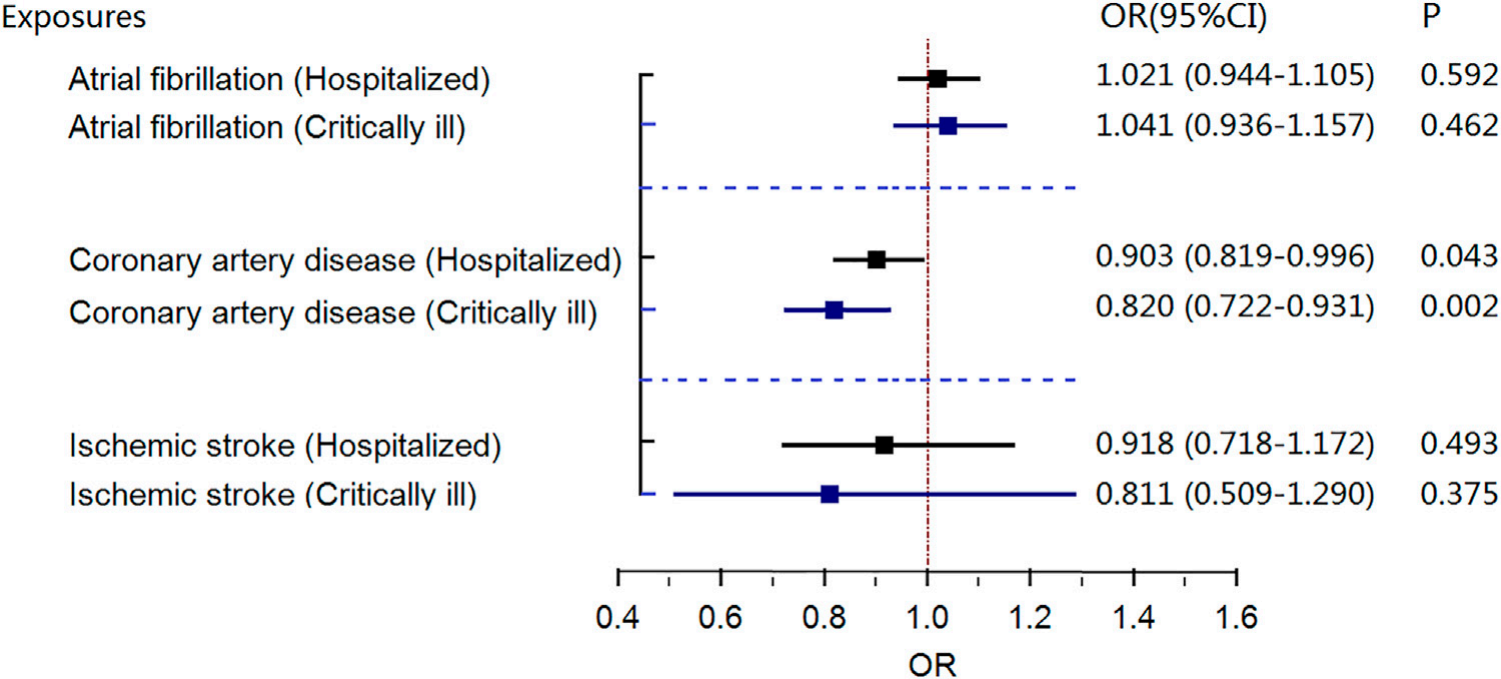
Results of the random-effects MR IVW model investigating the causal association between gene-predicted three cardiovascular diseases and risk of severe coronavirus disease 2019 (COVID-19) in COVID-19 GWAS data (without UKBB data). CI, confidence interval; OR, odds ratio; MR, Mendelian randomization; COVID-19, severe coronavirus disease 2019.

Genetically predicted coronary artery disease was associated with a lower risk of critically ill COVID-19, with an OR of 0.860 (random-effects MR IVW model, 95% CI 0.760–0.973; *p* = .017, Figure 3) in the GWAS meta-analysis and an OR of 0.820 (random-effects MR IVW model, 95% CI 0.722–0.931; *p* = .002, Figure 4) in GWAS meta-analysis (without the UKBB data). There was no evidence supporting an association of atrial fibrillation or ischemic stroke with the risk of critical COVID-19 or hospitalization with COVID-19.

### Sensitivity Analysis for Our MR

The OR estimates of the weighted median analysis (Supplementary Table S11 and Supplementary Table S12) were similar to those of the standard MR analysis (inverse-variance weighted method) but had low precision. The MR-Egger analysis for most outcomes revealed consistent estimates but had lower precision and did not indicate directional pleiotropy in the estimate of the association between genetically predicted hospitalized COVID-19 and ischemic stroke (Supplementary Table S11 and Supplementary Table S12). However, the indication of directional pleiotropy was observed in the estimate of the association between genetically predicted coronary artery disease and lower risk of critical COVID-19.

## Discussion

The current COVID-19 pandemic is rapidly evolving as a major threat to global health. Recent studies have explicitly described the pathogenesis, clinical characteristics, and complications of patients with COVID-19 in the acute phase (Cevik et al., 2020; Wiersinga et al., 2020), even the relatively long-term consequences of this severe illness (Huang et al., 2021), including the observational association between COVID-19 and several cardiovascular diseases (Azevedo et al., 2021). However, whether there is a causal association between COVID-19 and cardiovascular diseases is unclear.

When compared with traditional observational epidemiological studies, MR analysis may provide potential evidence to assess the causal association between COVID-19 and cardiovascular disease. Based on summary statistics from the largest and most updated GWAS data for COVID-19 in four different databases, our study provided suggestive evidence that genetically determined hospitalized COVID-19 is causally associated with a 4.1%–4.9% increased risk of ischemic stroke. It was reported that the incidence of cerebrovascular disease in patients who have suffered from severe infection was approximately 5.7% (Mao et al., 2020), and approximately 5% of COVID-19 patients later developed stroke with a median age of 71.6 years (Li et al., 2020). Elevated levels of CRP and D-dimer were observed in these patients (Li et al., 2020). Therefore, we speculate that the shared SNPs may contribute to abnormalities with the coagulation cascade and high inflammatory state, which may play an important role in the occurrence of ischemic stroke in patients with COVID-19 infections.

Interestingly, our study did not support causal effects for the observed association between genetically determined coronary artery disease and critical COVID-19. In contrast, our results showed that patients with coronary artery disease have a lower risk in developing critical COVID-19. Thus, there might be other mechanisms rather than genetics playing an important role in heightened susceptibility to COVID-19 for patients with coronary artery disease. For example, the heightened susceptibility to COVID-19 for patients with coronary artery disease may result from low immunity of the body because of indirect or direct injury by O_2_ supply–demand imbalance or inflammatory damage (Guo et al., 2020; Inciardi et al., 2020). Markedly, although the estimate of genetic association may be due to horizontal pleiotropy, causality cannot be excluded. Our estimates can explain the lifelong average effects of genetic variants, which cannot be fully interpreted in the same way as the effects from an observational study or within a briefer period observation. Moreover, the potential importance of a factor may also exist within shorter time frames even though no causal association was observed in our results, and further investigation may be needed to find relevant discrepancies.

A chief strength of the present study is that we assessed the causal associations between COVID-19 and three cardio-cerebrovascular diseases in the same study population using the MR method. Given that alleles are randomly assorted and fixed at conception, biases caused by confounding and reverse causality would not have been observed in our MR analysis. Hence, our results represent the lifetime effect between cardiovascular diseases and COVID-19. A further strength is that the cardiovascular disease GWAS data in our study was finished just in European ancestry populations, which could reduce bias due to population stratification. Therefore, potential confounders were small in our study. Pleiotropy is a potential limitation of MR analysis, which means that a genetic variant may be associated with more than one phenotype. Fortunately, no evidence of directional pleiotropy was found in our present study. Even the effect size is quite modest, it is estimated that there are many patients at risk of ischemic stroke, especially for the reason of huge COVID-19 patients. A potential limitation of our study is that only a few cases were enrolled in some outcomes. Therefore, weak associations due to insufficient power may have been overlooked.

In conclusion, using MR analysis, we found potential evidence about the causal effect of COVID-19 on the increased risk of ischemic stroke. Besides, other factors, rather than genetics, potentially contribute to the risk of coronary artery disease in patients with COVID-19.

## Data Availability

The original contributions presented in the study are included in the article/Supplementary Material, further inquiries can be directed to the corresponding author.
