# Supplementary material for: Genetic Predispositions Between COVID-19 and Three Cardio-Cerebrovascular Diseases
Source: Front Genet. 2022 Mar 16;13:743905. doi: 10.3389/fgene.2022.743905 (PMC8966609; doi:10.3389/fgene.2022.743905)
Supplement: Supplementary file 1 [file DataSheet1.docx]

**Supplementary material online:**

**Table S1. Characteristics of GWASs in atrial fibrillation, ischemic stroke and coronary artery disease.**

| **Characteristics** | **Atrial fibrillation** | **Coronary artery disease** | **Ischemic stroke** |
| --- | --- | --- | --- |
| No. of controls | 482,295 | 261,984 | 406,111 |
| No. of cases | 55,114 | 34,541 | 34,217 |
| Sample size | 537409 | 296525 | 440328 |
| Year of publication | 2018 | 2017 | 2018 |
| Number of SNPs | 12,095,506 | 7,904,237 | 7,537,579 |
| Build | HG19/GRCh37 | HG19/GRCh37 | HG19/GRCh37 |
| Study population | European ancestry | European ancestry | European ancestry |

**Table S2. Contributing studies of the very severe respiratory confirmed COVID-19 GWAS data in European ancestry.**

| **Full name** | **n_cases** | **n_controls** |
| --- | --- | --- |
| BQC19_EUR | 88 | 552 |
| BelCovid_EUR | 182 | 1477 |
| CU_EUR | 203 | 2149 |
| FinnGen_FIN | 68 | 238643 |
| GENCOVID_EUR | 724 | 2443 |
| GHS_Freeze_145_EUR | 53 | 112862 |
| SweCovid_EUR | 77 | 3748 |
| idipaz24genetics_EUR | 59 | 75 |
| Amsterdam_UMC_COVID_study_group_EUR | 66 | 1413 |
| SPGRX_EUR | 101 | 302 |
| genomicc_EUR | 1676 | 8380 |
| Italy_HOSTAGE_EUR | 698 | 1255 |
| Spain_HOSTAGE_EUR | 302 | 925 |
| 23ANDME_EUR | 495 | 680440 |
| UKBB_EUR | 309 | 328577 |

**Abbreviations:** GWAS = genome-wide association study, COVID-19 = coronavirus disease 2019, BQC19 = Biobanque Quebec COVID19, BelCovid = Genetic modifiers for COVID-19 related illness, CU = Columbia University COVID19 Biobank, GHS_Freeze_145 = Geisinger Health System, SweCovid = The genetic predisposition to severe COVID-19, idipaz24genetics = 24Genetics, SPGRX = Determining the Molecular Pathways and Genetic Predisposition of the Acute Inflammatory Process Caused by SARS-CoV-2, Italy_HOSTAGE = Italy COVID19-Host(a)ge, Spain_HOSTAGE_EUR = Spain COVID19-Host(a)ge.

**Table S3. Contributing studies of the hospitalized COVID-19 GWAS data in European ancestry.**

| **Full name** | **n_cases** | **n_controls** |
| --- | --- | --- |
| BelCovid_EUR | 361 | 122 |
| BoSCO_EUR | 212 | 512 |
| EstBB_EUR | 60 | 512 |
| FHoGID_EUR | 362 | 259 |
| FinnGen_FIN | 106 | 520 |
| GENCOVID_EUR | 892 | 249 |
| UCLA_EUR | 80 | 123 |
| UKBB_EUR | 1670 | 4610 |
| SPGRX_EUR | 311 | 51 |
| DECODE_EUR | 89 | 1808 |
| MVP_EUR | 436 | 1083 |
| Ancestry_EUR | 250 | 1967 |

**Abbreviations:** GWAS = genome-wide association study, COVID-19 = coronavirus disease 2019, BelCovid = Genetic modifiers for COVID-19 related illness, BoSCO = Bonn Study of COVID19 genetics, EstBB = Estonian Biobank, UCLA = UCLA Precision Health COVID-19 Biobank, UKBB = UK Biobank, SPGRX = Determining the Molecular Pathways and Genetic Predisposition of the Acute Inflammatory Process Caused by SARS-CoV-2, MVP = Million Veterans Program.

**Table S4.**  List of Genetic Instruments for critically ill COVID19 by Each Instrumental SNPs (GWAS Significance with P < 5×10^−7^ and linkage disequilibrium threshold with R^2^ < 0.001)

| **No.** | **SNP** | **Chr.** | **EA** | **OA** | **EAF** | **β** | **SE** |
| --- | --- | --- | --- | --- | --- | --- | --- |
| 1 | rs13050728 | 21 | C | T | 0.663 | -0.200 | 0.029 |
| 2 | rs2109069 | 19 | A | G | 0.329 | 0.257 | 0.028 |
| 3 | rs77534576 | 17 | T | C | 0.035 | 0.460 | 0.075 |
| 4 | rs7135260 | 12 | C | T | 0.674 | 0.192 | 0.028 |
| 5 | rs2510989 | 11 | T | A | 0.534 | -0.182 | 0.034 |
| 6 | rs2237698 | 7 | T | C | 0.090 | 0.237 | 0.040 |
| 7 | rs622568 | 7 | C | A | 0.146 | 0.226 | 0.037 |
| 8 | rs111837807 | 6 | C | T | 0.100 | 0.295 | 0.043 |

Chr. indicates chromosome; EA, effect allele; OA, other allele; EAF, effect allele frequency.

**Table S5.**  List of Genetic Instruments for critically ill COVID19 (leave UKBB) by Each Instrumental SNPs (GWAS Significance with P < 5×10^−7^ and linkage disequilibrium threshold with R^2^ < 0.001)

| **No.** | **SNP** | **Chr.** | **EA** | **OA** | **EAF** | **β** | **SE** |
| --- | --- | --- | --- | --- | --- | --- | --- |
| 1 | rs2834163 | 21 | A | G | 0.639 | -0.189 | 0.030 |
| 2 | rs2109069 | 19 | A | G | 0.333 | 0.276 | 0.030 |
| 3 | rs77534576 | 17 | T | C | 0.041 | 0.467 | 0.079 |
| 4 | rs10735079 | 12 | A | G | 0.695 | 0.211 | 0.029 |
| 5 | rs2237698 | 7 | T | C | 0.097 | 0.239 | 0.041 |
| 6 | rs622568 | 7 | C | A | 0.167 | 0.255 | 0.039 |
| 7 | rs111837807 | 6 | C | T | 0.130 | 0.318 | 0.044 |
| 8 | rs35081325 | 3 | T | A | 0.081 | 0.625 | 0.046 |

Chr. indicates chromosome; EA, effect allele; OA, other allele; EAF, effect allele frequency.

**Table S6.**  List of Genetic Instruments for hospitalized COVID19 by Each Instrumental SNPs (GWAS Significance with P < 5×10^−7^ and linkage disequilibrium threshold with R^2^ < 0.001)

| **No.** | **SNP** | **Chr.** | **EA** | **OA** | **EAF** | **β** | **SE** |
| --- | --- | --- | --- | --- | --- | --- | --- |
| 1 | rs13050728 | 21 | C | T | 0.653 | -0.168 | 0.020 |
| 2 | rs2109069 | 19 | A | G | 0.323 | 0.151 | 0.020 |
| 3 | rs2660 | 12 | A | G | 0.690 | 0.116 | 0.019 |
| 4 | rs622568 | 7 | C | A | 0.151 | 0.154 | 0.026 |
| 5 | rs111837807 | 6 | C | T | 0.091 | 0.165 | 0.031 |
| 6 | rs35081325 | 3 | T | A | 0.081 | 0.488 | 0.032 |
| 7 | rs41264915 | 1 | G | A | 0.093 | -0.162 | 0.030 |

Chr. indicates chromosome; EA, effect allele; OA, other allele; EAF, effect allele frequency.

**Table S7.**  List of Genetic Instruments for hospitalized COVID19 (leave UKBB and 23andme) by Each Instrumental SNPs (GWAS Significance with P < 5×10^−7^ and linkage disequilibrium threshold with R^2^ < 0.001)

| **No.** | **SNP** | **Chr.** | **EA** | **OA** | **EAF** | **β** | **SE** |
| --- | --- | --- | --- | --- | --- | --- | --- |
| 1 | rs13050728 | 21 | C | T | 0.638 | -0.186 | 0.024 |
| 2 | rs2109069 | 19 | A | G | 0.322 | 0.187 | 0.024 |
| 3 | rs1859330 | 12 | A | G | 0.698 | 0.156 | 0.023 |
| 4 | rs622568 | 7 | C | A | 0.178 | 0.178 | 0.030 |
| 5 | rs111837807 | 6 | C | T | 0.100 | 0.216 | 0.034 |
| 6 | rs35081325 | 3 | T | A | 0.086 | 0.546 | 0.035 |
| 7 | rs41264915 | 1 | G | A | 0.082 | -0.205 | 0.036 |
| 8 | rs13050728 | 21 | C | T | 0.638 | -0.186 | 0.024 |

Chr. indicates chromosome; EA, effect allele; OA, other allele; EAF, effect allele frequency.

**Table S8.**  List of Genetic Instruments for atrial fibrillation by Each Instrumental SNPs (GWAS Significance with P < 5×10^−8^ and linkage disequilibrium threshold with R^2^ < 0.001)

| **No.** | **SNP** | **Chr.** | **EA** | **OA** | **EAF** | **β** | **SE** |
| --- | --- | --- | --- | --- | --- | --- | --- |
| 1 | rs74832855 | 1 | G | A | NA | 0.143 | 0.020 |
| 2 | rs4484922 | 1 | C | G | NA | -0.063 | 0.008 |
| 3 | rs2885697 | 1 | T | G | NA | -0.039 | 0.008 |
| 4 | rs11264280 | 1 | T | C | NA | 0.127 | 0.008 |
| 5 | rs2813865 | 1 | G | A | NA | 0.048 | 0.009 |
| 6 | rs10753933 | 1 | G | T | NA | -0.074 | 0.007 |
| 7 | rs146518726 | 1 | A | G | NA | 0.162 | 0.025 |
| 8 | rs79187193 | 1 | A | G | NA | -0.112 | 0.018 |
| 9 | rs7549338 | 1 | G | C | NA | -0.045 | 0.007 |
| 10 | rs72700114 | 1 | C | G | NA | 0.203 | 0.014 |
| 11 | rs577676 | 1 | T | C | NA | -0.096 | 0.007 |
| 12 | rs880315 | 1 | C | T | NA | 0.044 | 0.008 |
| 13 | rs2372992 | 2 | G | A | NA | -0.040 | 0.008 |
| 14 | rs35504893 | 2 | T | C | NA | 0.090 | 0.009 |
| 15 | rs10165883 | 2 | T | C | NA | -0.064 | 0.007 |
| 16 | rs2540949 | 2 | T | A | NA | -0.075 | 0.007 |
| 17 | rs35215597 | 2 | G | A | NA | -0.076 | 0.009 |
| 18 | rs6546620 | 2 | C | T | NA | 0.071 | 0.009 |
| 19 | rs295114 | 2 | T | C | NA | -0.068 | 0.007 |
| 20 | rs62197371 | 2 | A | C | NA | -0.040 | 0.008 |
| 21 | rs12992412 | 2 | T | A | NA | 0.041 | 0.007 |
| 22 | rs4672423 | 2 | T | C | NA | -0.043 | 0.007 |
| 23 | rs72926475 | 2 | A | G | NA | -0.071 | 0.011 |
| 24 | rs9872035 | 3 | T | C | NA | -0.037 | 0.007 |
| 25 | rs7632427 | 3 | C | T | NA | -0.043 | 0.007 |
| 26 | rs4855075 | 3 | T | C | NA | 0.060 | 0.010 |
| 27 | rs73032363 | 3 | G | A | NA | -0.043 | 0.008 |
| 28 | rs3856795 | 3 | A | G | NA | 0.040 | 0.008 |
| 29 | rs3922843 | 3 | G | A | NA | -0.047 | 0.008 |
| 30 | rs2306272 | 3 | C | T | NA | 0.051 | 0.008 |
| 31 | rs17490701 | 3 | A | G | NA | -0.070 | 0.011 |
| 32 | rs6810325 | 3 | C | T | NA | 0.075 | 0.008 |
| 33 | rs6790396 | 3 | G | C | NA | 0.064 | 0.007 |
| 34 | rs10213171 | 4 | G | C | NA | 0.104 | 0.014 |
| 35 | rs79835095 | 4 | T | C | NA | 0.045 | 0.009 |
| 36 | rs2595107 | 4 | C | T | NA | 0.143 | 0.008 |
| 37 | rs6841049 | 4 | G | T | NA | -0.037 | 0.007 |
| 38 | rs10520260 | 4 | G | A | NA | -0.054 | 0.008 |
| 39 | rs3822259 | 4 | T | G | NA | 0.046 | 0.008 |
| 40 | rs6838973 | 4 | T | C | NA | -0.184 | 0.007 |
| 41 | rs11099696 | 4 | T | C | NA | -0.038 | 0.007 |
| 42 | rs4307025 | 4 | A | T | NA | 0.289 | 0.008 |
| 43 | rs34750263 | 5 | T | C | NA | 0.087 | 0.008 |
| 44 | rs174048 | 5 | C | T | NA | 0.067 | 0.010 |
| 45 | rs78758741 | 5 | C | T | NA | 0.061 | 0.012 |
| 46 | rs6882776 | 5 | A | G | NA | -0.060 | 0.008 |
| 47 | rs115219487 | 5 | C | T | NA | 0.066 | 0.013 |
| 48 | rs716845 | 5 | A | G | NA | 0.059 | 0.008 |
| 49 | rs17118812 | 5 | C | T | NA | 0.040 | 0.008 |
| 50 | rs12208899 | 6 | A | G | NA | 0.049 | 0.009 |
| 51 | rs9481842 | 6 | G | T | NA | 0.066 | 0.008 |
| 52 | rs73366713 | 6 | A | G | NA | -0.105 | 0.011 |
| 53 | rs12211255 | 6 | A | C | NA | 0.059 | 0.012 |
| 54 | rs13191450 | 6 | C | A | NA | -0.070 | 0.008 |
| 55 | rs117984853 | 6 | T | G | NA | 0.113 | 0.014 |
| 56 | rs34969716 | 6 | A | G | NA | 0.088 | 0.008 |
| 57 | rs6907805 | 6 | T | G | NA | -0.041 | 0.007 |
| 58 | rs112974895 | 6 | C | A | NA | -0.058 | 0.012 |
| 59 | rs3176326 | 6 | A | G | NA | -0.060 | 0.009 |
| 60 | rs1307274 | 6 | G | T | NA | -0.074 | 0.014 |
| 61 | rs62483627 | 7 | A | G | NA | 0.049 | 0.008 |
| 62 | rs55985730 | 7 | G | T | NA | 0.096 | 0.017 |
| 63 | rs11773845 | 7 | A | C | NA | 0.116 | 0.007 |
| 64 | rs11768850 | 7 | T | C | NA | 0.039 | 0.007 |
| 65 | rs1182197 | 7 | C | A | NA | -0.037 | 0.007 |
| 66 | rs55734480 | 7 | A | G | NA | 0.050 | 0.008 |
| 67 | rs7789146 | 7 | A | G | NA | -0.057 | 0.009 |
| 68 | rs3731326 | 7 | G | A | NA | -0.046 | 0.008 |
| 69 | rs74910854 | 7 | G | A | NA | 0.094 | 0.016 |
| 70 | rs6462078 | 7 | A | C | NA | 0.058 | 0.009 |
| 71 | rs62521286 | 8 | G | A | NA | 0.122 | 0.015 |
| 72 | rs10903345 | 8 | G | A | NA | -0.038 | 0.008 |
| 73 | rs28372085 | 8 | T | C | NA | -0.093 | 0.012 |
| 74 | rs35006907 | 8 | A | C | NA | 0.045 | 0.008 |
| 75 | rs6993266 | 8 | A | G | NA | 0.044 | 0.007 |
| 76 | rs7508 | 8 | A | G | NA | 0.072 | 0.008 |
| 77 | rs4743034 | 9 | A | G | NA | 0.049 | 0.008 |
| 78 | rs4842131 | 9 | C | T | NA | 0.039 | 0.007 |
| 79 | rs35991181 | 9 | C | G | NA | -0.042 | 0.008 |
| 80 | rs10760361 | 9 | T | G | NA | -0.043 | 0.008 |
| 81 | rs4385527 | 9 | A | G | NA | 0.092 | 0.007 |
| 82 | rs4977397 | 9 | G | A | NA | -0.043 | 0.008 |
| 83 | rs1044258 | 10 | C | T | NA | -0.046 | 0.008 |
| 84 | rs2296610 | 10 | T | G | NA | 0.172 | 0.033 |
| 85 | rs7919685 | 10 | T | G | NA | -0.058 | 0.007 |
| 86 | rs11001667 | 10 | G | A | NA | 0.062 | 0.009 |
| 87 | rs11598047 | 10 | G | A | NA | 0.153 | 0.010 |
| 88 | rs80056983 | 10 | T | C | NA | 0.122 | 0.010 |
| 89 | rs60212594 | 10 | C | G | NA | -0.110 | 0.010 |
| 90 | rs1765131 | 11 | C | G | NA | -0.038 | 0.008 |
| 91 | rs76097649 | 11 | A | G | NA | 0.126 | 0.014 |
| 92 | rs949078 | 11 | T | C | NA | -0.053 | 0.008 |
| 93 | rs1822273 | 11 | A | G | NA | -0.068 | 0.008 |
| 94 | rs113819537 | 12 | G | C | NA | -0.049 | 0.008 |
| 95 | rs1454933 | 12 | C | A | NA | 0.063 | 0.011 |
| 96 | rs883079 | 12 | T | C | NA | 0.120 | 0.008 |
| 97 | rs11057400 | 12 | T | C | NA | -0.046 | 0.008 |
| 98 | rs11180703 | 12 | A | G | NA | -0.046 | 0.007 |
| 99 | rs35349325 | 12 | C | T | NA | -0.052 | 0.007 |
| 100 | rs12810346 | 12 | T | C | NA | 0.066 | 0.011 |
| 101 | rs10842383 | 12 | T | C | NA | -0.109 | 0.010 |
| 102 | rs57671871 | 12 | C | T | NA | 0.071 | 0.014 |
| 103 | rs7978685 | 12 | C | T | NA | -0.055 | 0.008 |
| 104 | rs11835327 | 12 | G | A | NA | 0.068 | 0.012 |
| 105 | rs9580438 | 13 | C | T | NA | 0.057 | 0.008 |
| 106 | rs8005490 | 14 | C | T | NA | -0.047 | 0.007 |
| 107 | rs2145587 | 14 | A | G | NA | 0.075 | 0.008 |
| 108 | rs2738413 | 14 | G | A | NA | -0.081 | 0.007 |
| 109 | rs10873299 | 14 | G | A | NA | -0.048 | 0.008 |
| 110 | rs28631169 | 14 | T | C | NA | 0.070 | 0.009 |
| 111 | rs12908437 | 15 | C | T | NA | -0.047 | 0.007 |
| 112 | rs12591736 | 15 | A | G | NA | -0.061 | 0.010 |
| 113 | rs74022964 | 15 | T | C | NA | 0.106 | 0.010 |
| 114 | rs2286466 | 16 | G | A | NA | 0.072 | 0.010 |
| 115 | rs11075959 | 16 | G | A | NA | 0.140 | 0.022 |
| 116 | rs2359171 | 16 | A | T | NA | 0.188 | 0.009 |
| 117 | rs8073937 | 17 | A | G | NA | -0.050 | 0.007 |
| 118 | rs72811294 | 17 | C | G | NA | -0.067 | 0.012 |
| 119 | rs12942576 | 17 | C | T | NA | -0.040 | 0.008 |
| 120 | rs7219869 | 17 | G | C | NA | 0.046 | 0.007 |
| 121 | rs242557 | 17 | A | G | NA | -0.044 | 0.008 |
| 122 | rs2230234 | 18 | G | A | NA | 0.070 | 0.013 |
| 123 | rs9953366 | 18 | C | T | NA | 0.050 | 0.008 |
| 124 | rs2974231 | 19 | A | G | NA | 0.039 | 0.008 |
| 125 | rs2145274 | 20 | C | A | NA | -0.102 | 0.014 |
| 126 | rs7269123 | 20 | T | C | NA | -0.044 | 0.008 |
| 127 | rs2834618 | 21 | G | T | NA | -0.110 | 0.013 |
| 128 | rs361834 | 22 | A | G | NA | -0.047 | 0.008 |

Chr. indicates chromosome; EA, effect allele; OA, other allele; EAF, effect allele frequency.

**Table S9.**  List of Genetic Instruments for coronary artery disease by Each Instrumental SNPs (GWAS Significance with P < 5×10^−8^ and linkage disequilibrium threshold with R^2^ < 0.001)

| **No.** | **SNP** | **Chr.** | **EA** | **OA** | **EAF** | **β** | **SE** |
| --- | --- | --- | --- | --- | --- | --- | --- |
| 1 | rs4072980 | 1 | A | G | 0.430 | -0.033 | 0.005 |
| 2 | rs11591147 | 1 | T | G | 0.016 | -0.241 | 0.025 |
| 3 | rs10858079 | 1 | G | A | 0.186 | -0.037 | 0.007 |
| 4 | rs17114046 | 1 | G | A | 0.094 | -0.096 | 0.009 |
| 5 | rs35158675 | 1 | A | G | 0.682 | 0.070 | 0.006 |
| 6 | rs36096196 | 1 | T | C | 0.148 | 0.047 | 0.008 |
| 7 | rs2493298 | 1 | A | C | 0.136 | 0.051 | 0.008 |
| 8 | rs12143614 | 1 | A | T | 0.108 | 0.057 | 0.009 |
| 9 | rs1892094 | 1 | T | C | 0.496 | -0.036 | 0.005 |
| 10 | rs6700559 | 1 | T | C | 0.472 | -0.028 | 0.005 |
| 11 | rs602633 | 1 | G | T | 0.766 | 0.100 | 0.006 |
| 12 | rs4845625 | 1 | C | T | 0.566 | -0.040 | 0.005 |
| 13 | rs2820315 | 1 | T | C | 0.301 | 0.036 | 0.006 |
| 14 | rs16986953 | 2 | A | G | 0.110 | 0.081 | 0.010 |
| 15 | rs6728861 | 2 | A | G | 0.117 | 0.105 | 0.009 |
| 16 | rs1250229 | 2 | C | T | 0.728 | -0.044 | 0.006 |
| 17 | rs515135 | 2 | C | T | 0.816 | 0.056 | 0.007 |
| 18 | rs11677932 | 2 | A | G | 0.316 | -0.034 | 0.006 |
| 19 | rs582384 | 2 | A | C | 0.525 | 0.033 | 0.006 |
| 20 | rs2252641 | 2 | C | T | 0.470 | 0.037 | 0.005 |
| 21 | rs6544713 | 2 | C | T | 0.696 | -0.049 | 0.006 |
| 22 | rs12999907 | 2 | G | A | 0.181 | -0.048 | 0.007 |
| 23 | rs2571445 | 2 | G | A | 0.611 | -0.037 | 0.005 |
| 24 | rs6743030 | 2 | T | C | 0.465 | 0.057 | 0.006 |
| 25 | rs7617773 | 3 | T | C | 0.669 | 0.036 | 0.005 |
| 26 | rs56210800 | 3 | G | C | 0.126 | 0.063 | 0.008 |
| 27 | rs789294 | 3 | G | C | 0.835 | 0.064 | 0.008 |
| 28 | rs12897 | 3 | A | G | 0.591 | -0.036 | 0.006 |
| 29 | rs73079003 | 3 | A | G | 0.118 | -0.051 | 0.009 |
| 30 | rs7621025 | 3 | C | T | 0.753 | 0.045 | 0.006 |
| 31 | rs185244 | 3 | T | C | 0.163 | 0.064 | 0.008 |
| 32 | rs34991912 | 3 | C | T | 0.566 | -0.038 | 0.006 |
| 33 | rs4266144 | 3 | G | C | 0.323 | 0.035 | 0.006 |
| 34 | rs4678145 | 3 | C | G | 0.128 | 0.064 | 0.008 |
| 35 | rs10512861 | 3 | T | G | 0.143 | -0.043 | 0.007 |
| 36 | rs10857147 | 4 | T | A | 0.279 | 0.049 | 0.006 |
| 37 | rs2880099 | 4 | C | A | 0.866 | -0.055 | 0.009 |
| 38 | rs7692387 | 4 | A | G | 0.189 | -0.064 | 0.007 |
| 39 | rs17081933 | 4 | A | T | 0.198 | 0.040 | 0.007 |
| 40 | rs7678555 | 4 | C | A | 0.283 | 0.049 | 0.006 |
| 41 | rs7696431 | 4 | G | T | 0.490 | -0.031 | 0.006 |
| 42 | rs12500824 | 4 | G | A | 0.644 | -0.034 | 0.005 |
| 43 | rs6841581 | 4 | A | G | 0.156 | 0.075 | 0.007 |
| 44 | rs3775058 | 4 | T | A | 0.766 | -0.039 | 0.007 |
| 45 | rs112941079 | 5 | G | A | 0.132 | -0.062 | 0.009 |
| 46 | rs3936511 | 5 | G | A | 0.177 | 0.037 | 0.007 |
| 47 | rs246600 | 5 | T | C | 0.463 | 0.043 | 0.005 |
| 48 | rs273909 | 5 | G | A | 0.125 | 0.049 | 0.008 |
| 49 | rs74618856 | 6 | C | T | 0.559 | 0.033 | 0.006 |
| 50 | rs12212146 | 6 | C | T | 0.067 | -0.092 | 0.012 |
| 51 | rs9349379 | 6 | G | A | 0.409 | 0.107 | 0.006 |
| 52 | rs1591805 | 6 | G | A | 0.507 | -0.037 | 0.006 |
| 53 | rs2814944 | 6 | A | G | 0.155 | 0.048 | 0.007 |
| 54 | rs4613862 | 6 | C | A | 0.469 | -0.032 | 0.005 |
| 55 | rs17478367 | 6 | C | G | 0.141 | 0.046 | 0.008 |
| 56 | rs2327429 | 6 | C | T | 0.305 | -0.066 | 0.006 |
| 57 | rs146534110 | 6 | T | G | 0.013 | 0.184 | 0.026 |
| 58 | rs117733303 | 6 | G | A | 0.019 | 0.429 | 0.024 |
| 59 | rs733701 | 6 | T | C | 0.250 | 0.040 | 0.006 |
| 60 | rs17080091 | 6 | T | C | 0.084 | -0.054 | 0.009 |
| 61 | rs55730499 | 6 | T | C | 0.069 | 0.312 | 0.012 |
| 62 | rs6909752 | 6 | A | G | 0.355 | 0.044 | 0.006 |
| 63 | rs1321309 | 6 | A | G | 0.491 | 0.028 | 0.005 |
| 64 | rs6905288 | 6 | A | G | 0.581 | 0.039 | 0.006 |
| 65 | rs71566846 | 6 | T | C | 0.066 | 0.083 | 0.011 |
| 66 | rs3127580 | 6 | T | C | 0.142 | 0.084 | 0.008 |
| 67 | rs2107732 | 7 | A | G | 0.087 | -0.057 | 0.010 |
| 68 | rs975722 | 7 | G | A | 0.405 | 0.028 | 0.005 |
| 69 | rs11556924 | 7 | T | C | 0.362 | -0.055 | 0.006 |
| 70 | rs10237377 | 7 | T | G | 0.361 | -0.034 | 0.006 |
| 71 | rs3918226 | 7 | T | C | 0.073 | 0.107 | 0.012 |
| 72 | rs10267593 | 7 | A | G | 0.201 | -0.036 | 0.006 |
| 73 | rs2107595 | 7 | A | G | 0.177 | 0.075 | 0.007 |
| 74 | rs4724806 | 7 | C | G | 0.768 | 0.039 | 0.007 |
| 75 | rs2189839 | 7 | G | A | 0.710 | -0.035 | 0.006 |
| 76 | rs10093110 | 8 | A | G | 0.417 | -0.032 | 0.006 |
| 77 | rs6982502 | 8 | T | C | 0.530 | -0.050 | 0.005 |
| 78 | rs6984210 | 8 | G | C | 0.061 | 0.078 | 0.012 |
| 79 | rs17091891 | 8 | C | T | 0.129 | -0.059 | 0.008 |
| 80 | rs4977574 | 9 | G | A | 0.481 | 0.179 | 0.006 |
| 81 | rs944172 | 9 | T | C | 0.720 | -0.040 | 0.006 |
| 82 | rs885150 | 9 | C | T | 0.270 | 0.036 | 0.006 |
| 83 | rs9337951 | 10 | A | G | 0.318 | 0.054 | 0.006 |
| 84 | rs4752700 | 10 | G | A | 0.449 | 0.033 | 0.005 |
| 85 | rs61848342 | 10 | C | T | 0.365 | 0.036 | 0.006 |
| 86 | rs17680741 | 10 | C | T | 0.285 | -0.042 | 0.006 |
| 87 | rs1412444 | 10 | T | C | 0.353 | 0.056 | 0.006 |
| 88 | rs1870634 | 10 | G | T | 0.649 | 0.060 | 0.006 |
| 89 | rs3740390 | 10 | T | C | 0.107 | -0.066 | 0.008 |
| 90 | rs4918072 | 10 | A | G | 0.271 | 0.039 | 0.006 |
| 91 | rs12801636 | 11 | A | G | 0.236 | -0.040 | 0.006 |
| 92 | rs7926712 | 11 | G | A | 0.690 | 0.035 | 0.006 |
| 93 | rs7116641 | 11 | G | T | 0.312 | 0.031 | 0.006 |
| 94 | rs606452 | 11 | C | A | 0.829 | 0.047 | 0.007 |
| 95 | rs607562 | 11 | G | T | 0.715 | 0.038 | 0.006 |
| 96 | rs974819 | 11 | C | T | 0.692 | -0.061 | 0.006 |
| 97 | rs651821 | 11 | T | C | 0.901 | -0.069 | 0.010 |
| 98 | rs11601507 | 11 | A | C | 0.074 | 0.078 | 0.011 |
| 99 | rs2681492 | 12 | C | T | 0.186 | 0.056 | 0.007 |
| 100 | rs11838267 | 12 | C | T | 0.132 | -0.051 | 0.008 |
| 101 | rs11170820 | 12 | G | C | 0.071 | 0.083 | 0.012 |
| 102 | rs10841443 | 12 | G | C | 0.659 | 0.046 | 0.006 |
| 103 | rs11107903 | 12 | A | G | 0.077 | -0.075 | 0.011 |
| 104 | rs7137828 | 12 | T | C | 0.554 | -0.064 | 0.006 |
| 105 | rs11057841 | 12 | T | C | 0.150 | 0.062 | 0.007 |
| 106 | rs1169288 | 12 | C | A | 0.328 | 0.049 | 0.006 |
| 107 | rs9515203 | 13 | C | T | 0.261 | -0.060 | 0.006 |
| 108 | rs9319428 | 13 | A | G | 0.312 | 0.036 | 0.006 |
| 109 | rs12873154 | 13 | G | A | 0.126 | 0.064 | 0.008 |
| 110 | rs3783324 | 14 | C | T | 0.191 | 0.041 | 0.007 |
| 111 | rs2145598 | 14 | A | G | 0.576 | -0.028 | 0.005 |
| 112 | rs7145159 | 14 | C | T | 0.477 | 0.032 | 0.005 |
| 113 | rs8003602 | 14 | C | T | 0.732 | 0.054 | 0.007 |
| 114 | rs56225615 | 15 | T | C | 0.150 | 0.058 | 0.008 |
| 115 | rs1807214 | 15 | C | A | 0.111 | -0.064 | 0.010 |
| 116 | rs6494488 | 15 | A | G | 0.812 | 0.038 | 0.007 |
| 117 | rs72743461 | 15 | A | C | 0.221 | -0.058 | 0.007 |
| 118 | rs7173743 | 15 | C | T | 0.446 | -0.064 | 0.005 |
| 119 | rs17514846 | 15 | A | C | 0.461 | 0.056 | 0.005 |
| 120 | rs7500448 | 16 | G | A | 0.241 | -0.056 | 0.007 |
| 121 | rs12149545 | 16 | A | G | 0.304 | -0.037 | 0.006 |
| 122 | rs7199941 | 16 | G | A | 0.602 | -0.037 | 0.005 |
| 123 | rs7188857 | 16 | G | A | 0.241 | -0.049 | 0.007 |
| 124 | rs11080107 | 17 | C | T | 0.485 | 0.036 | 0.006 |
| 125 | rs7206935 | 17 | T | C | 0.181 | 0.043 | 0.008 |
| 126 | rs170041 | 17 | T | C | 0.294 | -0.047 | 0.006 |
| 127 | rs62076439 | 17 | T | G | 0.341 | 0.048 | 0.006 |
| 128 | rs12936587 | 17 | A | G | 0.438 | -0.032 | 0.005 |
| 129 | rs9892152 | 17 | C | T | 0.528 | 0.033 | 0.005 |
| 130 | rs1962412 | 17 | C | T | 0.688 | 0.041 | 0.006 |
| 131 | rs9964304 | 18 | C | A | 0.284 | 0.038 | 0.006 |
| 132 | rs663640 | 18 | T | C | 0.222 | 0.038 | 0.007 |
| 133 | rs7412 | 19 | T | C | 0.079 | -0.137 | 0.011 |
| 134 | rs17616620 | 19 | A | G | 0.077 | 0.057 | 0.010 |
| 135 | rs7251815 | 19 | T | G | 0.216 | 0.051 | 0.007 |
| 136 | rs7256873 | 19 | A | G | 0.177 | -0.041 | 0.008 |
| 137 | rs55791371 | 19 | C | A | 0.113 | -0.116 | 0.009 |
| 138 | rs4803455 | 19 | A | C | 0.495 | -0.048 | 0.006 |
| 139 | rs11673093 | 19 | A | G | 0.257 | 0.043 | 0.007 |
| 140 | rs3827066 | 20 | T | C | 0.144 | 0.042 | 0.007 |
| 141 | rs260020 | 20 | T | C | 0.131 | 0.052 | 0.008 |
| 142 | rs867186 | 20 | G | A | 0.103 | -0.057 | 0.008 |
| 143 | rs28451064 | 21 | A | G | 0.124 | 0.108 | 0.009 |
| 144 | rs6001960 | 22 | G | A | 0.723 | -0.035 | 0.006 |
| 145 | rs62233066 | 22 | G | A | 0.027 | -0.106 | 0.018 |

Chr. indicates chromosome; EA, effect allele; OA, other allele; EAF, effect allele frequency.

**Table S10.**  List of Genetic Instruments for ischemic stroke by Each Instrumental SNPs (GWAS Significance with P < 5×10^−8^ and linkage disequilibrium threshold with R^2^ < 0.001)

| **No.** | **SNP** | **Chr.** | **EA** | **OA** | **EAF** | **β** | **SE** |
| --- | --- | --- | --- | --- | --- | --- | --- |
| 1 | rs1052053 | 1 | G | A | 0.401 | -0.058 | 0.009 |
| 2 | rs17035646 | 1 | A | G | 0.405 | 0.054 | 0.009 |
| 3 | rs6825454 | 4 | C | T | 0.308 | 0.056 | 0.009 |
| 4 | rs6847935 | 4 | T | A | 0.326 | 0.078 | 0.010 |
| 5 | rs11957829 | 5 | G | A | 0.176 | -0.072 | 0.012 |
| 6 | rs4959130 | 6 | A | G | 0.137 | 0.083 | 0.014 |
| 7 | rs2107595 | 7 | A | G | 0.226 | 0.076 | 0.010 |
| 8 | rs42039 | 7 | T | C | 0.228 | -0.066 | 0.011 |
| 9 | rs7859727 | 9 | T | C | 0.536 | 0.051 | 0.008 |
| 10 | rs2005108 | 11 | T | C | 0.128 | 0.080 | 0.015 |
| 11 | rs3184504 | 12 | C | T | 0.548 | -0.075 | 0.010 |
| 12 | rs35436 | 12 | T | C | 0.381 | -0.050 | 0.009 |
| 13 | rs7304841 | 12 | C | A | 0.407 | -0.048 | 0.009 |
| 14 | rs9526212 | 13 | G | A | 0.761 | 0.062 | 0.010 |
| 15 | rs4932370 | 15 | A | G | 0.333 | 0.052 | 0.009 |
| 16 | rs12445022 | 16 | A | G | 0.306 | 0.061 | 0.010 |
| 17 | rs9909858 | 17 | C | T | 0.188 | 0.089 | 0.016 |
| 18 | rs1053007 | 19 | G | A | 0.651 | 0.048 | 0.009 |

Chr. indicates chromosome; EA, effect allele; OA, other allele; EAF, effect allele frequency.

**Table S11.** Associations between genetically predicted Covid-19 and cardiovascular diseases in sensitivity analyses using the weighted median and MR-Egger methods.

|  | **Weighted median** | |  | **MR-Egger** | | | |  |
| --- | --- | --- | --- | --- | --- | --- | --- | --- |
| **Outcome** | **OR (95% CI)** | ***P* value** |  | **OR (95% CI)** | ***P* value** |  | **Intercept^a^** | ***P* value** |
| **Critically ill COVID19** |  |  |  |  |  |  |  |  |
| Atrial fibrillation | 1.001(0.966-1.037) | 0.681 |  | 1.063(0.971-1.163) | 0.188 |  | 0.981(0.958-1.005) | 0.106 |
| Coronary artery disease | 0.994(0.969-1.020) | 0.612 |  | 1.004(0.926-1.088) | 0.931 |  | 1.000(0.979-1.022) | 0.983 |
| Ischemic Stroke | 1.028(0.989-1.070) | 0.169 |  | 1.033(0.955-1.117) | 0.418 |  | 0.998(0.979-1.018) | 0.857 |
| **Critically ill COVID19 (leave UKBB)** |  |  |  |  |  |  |  |  |
| Atrial fibrillation | 1.003(0.970-1.037) | 0.880 |  | 1.045(0.946-1.155) | 0.388 |  | 0.986(0.958-1.016) | 0.345 |
| Coronary artery disease | 0.995(0.970-1.021) | 0.665 |  | 0.944(0.908-1.088) | 0.899 |  | 1.004(0.977-1.032) | 0.767 |
| Ischemic Stroke | 1.041(1.001-1.082) | 0.049 |  | 1.020(0.938-1.110) | 0.639 |  | 1.003(0.980-1.027) | 0.789 |
| **Hospitalized COVID19** |  |  |  |  |  |  |  |  |
| Atrial fibrillation | 1.017(0.970-1.066) | 0.496 |  | 1.047(0.918-1.194) | 0.492 |  | 0.991(0.966-1.017) | 0.481 |
| Coronary artery disease | 0.994(0.960-1.030) | 0.727 |  | 0.992(0.877-1.122) | 0.903 |  | 1.003(0.978-1.029) | 0.793 |
| Ischemic Stroke | 1.069(1.010-1.132) | 0.022 |  | 1.065(0.958-1.184) | 0.239 |  | 0.997(0.978-1.017) | 0.756 |
| **Hospitalized COVID19 (leave UKBB)** |  |  |  |  |  |  |  |  |
| Atrial fibrillation | 1.009(0.968-1.051) | 0.685 |  | 1.049(0.935-1.178) | 0.423 |  | 0.988(0.963-1.014) | 0.368 |
| Coronary artery disease | 0.994(0.963-1.026) | 0.681 |  | 0.995(0.885-1.119) | 0.931 |  | 1.003(0.976-1.031) | 0.837 |
| Ischemic Stroke | 1.059(1.008-1.112) | 0.021 |  | 1.066(0.967-1.176) | 0.205 |  | 0.994(0.973-1.016) | 0.603 |

CI, confidence interval; OR odds ratio.

^a^ The MR-Egger intercept quantifies the effect of directional pleiotropy. Values that significantly differ from zero provide evidence that the Covid-19-associated single-nucleotide polymorphisms may influence the outcome through other pathways than through Covid-19.

**Table S12.** Associations between genetically predicted cardiovascular diseases and Covid-19 in sensitivity analyses using the weighted median and MR-Egger methods

|  | **Weighted median** | |  | **MR-Egger** | | | |  |
| --- | --- | --- | --- | --- | --- | --- | --- | --- |
| **Outcome** | **OR (95% CI)** | ***P* value** |  | **OR (95% CI)** | ***P* value** |  | **Intercept^a^** | ***P* value** |
| **Critically ill COVID19** |  |  |  |  |  |  |  |  |
| Atrial fibrillation | 1.046(0.896-1.221) | 0.568 |  | 1.054(0.840-1.324) | 0.650 |  | 0.999(0.982-1.017) | 0.876 |
| Coronary artery disease | 0.824(0.682-0.997) | 0.047 |  | 0.684(0.523-0.895) | 0.006 |  | 1.014(1.000-1.028) | 0.060 |
| Ischemic Stroke | 0.754(0.476-1.196) | 0.232 |  | 1.543(0.128-18.601) | 0.733 |  | 0.967(0.830-1.126) | 0.659 |
| **Critically ill COVID19 (leave UKBB)** |  |  |  |  |  |  |  |  |
| Atrial fibrillation | 1.023(0.871-1.202) | 0.779 |  | 1.090(0.846-1.403) | 0.508 |  | 0.996(0.977-1.016) | 0.697 |
| Coronary artery disease | 0.785(0.639-0.964) | 0.021 |  | 0.630(0.477-0.832) | 0.001 |  | 1.016(1.000-1.032) | 0.038 |
| Ischemic Stroke | 0.789(0.484-1.285) | 0.341 |  | 1.024(0.063-16.727) | 0.987 |  | 0.985(0.831-1.168) | 0.867 |
| **Hospitalized COVID19** |  |  |  |  |  |  |  |  |
| Atrial fibrillation | 1.063(0.962-1.175) | 0.230 |  | 1.096(0.932-1.290) | 0.270 |  | 0.996(0.984-1.008) | 0.517 |
| Coronary artery disease | 0.936(0.811-1.080) | 0.367 |  | 0.850(0.710-1.018) | 0.077 |  | 1.008(0.998-1.018) | 0.122 |
| Ischemic Stroke | 1.011(0.762-1.341) | 0.937 |  | 1.925(0.543-6.829) | 0.311 |  | 0.963(0.890-1.041) | 0.336 |
| **Hospitalized COVID19 (leave UKBB)** |  |  |  |  |  |  |  |  |
| Atrial fibrillation | 1.030(0.911-1.166) | 0.633 |  | 1.106(0.917-1.335) | 0.291 |  | 0.993(0.979-1.007) | 0.359 |
| Coronary artery disease | 0.864(0.729-1.025) | 0.093 |  | 0.716(0.577-0.888) | 0.002 |  | 1.014(1.002-1.026) | 0.018 |
| Ischemic Stroke | 0.873(0.623-1.223) | 0.429 |  | 0.921(0.213-3.991) | 0.913 |  | 1.000(0.914-1.094) | 0.996 |

CI, confidence interval; OR odds ratio.

^a^The MR-Egger intercept quantifies the effect of directional pleiotropy. Values that significantly differ from zero provide evidence that the Covid-19-associated single-nucleotide polymorphisms may influence the outcome through other pathways than through Covid-19.
